# Does type 2 diabetes affect the efficacy of therapeutic exercises for degenerative lumbar spinal stenosis?

**DOI:** 10.1186/s12891-023-06305-0

**Published:** 2023-03-16

**Authors:** Tengbin Shi, Zhi Chen, Dingxiang Hu, Wenwen Li, Zhenyu Wang, Wenge Liu

**Affiliations:** 1 Department of Orthopedics, Fujian Medical University Union Hospital, Fuzhou, China; 2 School of Health, Fujian Medical University, 350108 Fuzhou, Fujian, China

**Keywords:** Lumbar spinal stenosis, Type 2 diabetes, Exercise therapy, Multifidus muscle, Fatty infiltration

## Abstract

**Design:**

Propensity-matched retrospective study.

**Objectives:**

To determine whether type 2 diabetes mellitus (T2D) would affect prognosis in patients with degenerative lumbar spinal stenosis (DLSS) who underwent therapeutic exercises.

**Methods:**

This study included consecutive patients with or without T2D who underwent therapeutic exercises for symptomatic DLSS from December 2018 to January 2020. Baseline demographics and clinical and radiological data were collected. The 2 groups of patients were further matched in a 1:1 fashion based on the propensity score, balancing the groups on pre-treatment factors including age, sex, leg and back pain, and low back disability. The primary outcomes included self-reported leg pain intensity (Numerical Rating Scale, NRS) and low back disability (Oswestry Disability Index, ODI) and the secondary outcomes included low back pain intensity and walking capacity (self-paced walking test, SPWT) were compared at baseline, 6 weeks, and 12 weeks.

**Results:**

Forty-one pairs of patients were selected by propensity matching. After 6-week therapeutic exercises, patients with T2D achieved a lower improvement in leg pain at 6 weeks (NRS leg change, 1.21 ± 0.40 vs. 1.78 ± 0.52, P = 0.021) and 12 weeks (NRS leg change, 1.52 ± 0.92 vs. 2.18 ± 0.96, P = 0.007) above minimal clinically important difference (MCID), with a significant Group × Time interactions (F_1,80_ = 16.32, p < 0.001, ηp2 = 0.053). However, the two groups showed no difference in the improvement of ODI, although the sample had significant improvements at 6 weeks (ODI change 3.02 [95% CI, 2.08 to 2.77], P < 0.001) and 12 weeks ([ODI change 3.82 [95% CI, 4.03 to 4.90], P < 0.001), 46% of the patients achieved an MCID.

**Conclusion:**

Six-week therapeutic exercises have an inferior effect on DLSS patients with T2D. Findings from this study will provide an increased understanding of exercise treatment in patients with DLSS.

**Supplementary Information:**

The online version contains supplementary material available at.10.1186/s12891-023-06305-0.

## Background

Degenerative lumbar spinal stenosis (DLSS) refers to a variety of factors causing degenerative changes in the lumbar spinal canal, nerve root canal, and lateral crypt, resulting in narrowing of the lumbar spinal luminal lumen and compression of blood vessels, nerve roots, and cauda equina, as well as the clinical syndromes described below, which are characterized by low back pain and neurogenic intermittent claudication [1, 2]. The negative impact of DLSS on the quality of life of the elderly is more serious than that of the hip, and knee degenerative diseases, and cardiovascular and cerebrovascular diseases [3]. The prevalence and economic burden of DLSS are expected to rise significantly as the population ages [4].

People are currently looking for the cause of degenerative lumbar spine disease, stating that metabolic factors and toxic environments can accelerate lumbar spine disease degeneration, so the correlation between metabolic diseases such as Type 2 diabetes mellitus (T2D for short) and lumbar spine disease has intrigued people’s interest [5–7]. According to reports, the global diabetes prevalence in 20-79-year-olds is expected to be 10.5% (536.6 million people) in 2021, rising to 12.2% (783.2 million) in 2045 [8]. Diabetes is a disease that affects multiple organs, including bones, cartilage, and intervertebral discs, and may be a predisposing factor for lumbar spinal stenosis [9]. Svenja’s [10] diabetes model showed pathological changes in the spinal structure that was similar to degenerative changes in human discs, such as a reduction in intervertebral disc height, a decrease in vertebral bone mass, and terminal plate hardening, among other things. T2D also had an impact on the surgical efficacy of degenerative lumbar spine diseases, leading to lower fusion rates and quality, poor pain improvement, and a higher incidence of postoperative complications [11–13].

However, non-surgical treatment is often the preferred option for patients with DLSS, especially those with mild to moderate DLSS, and a period of conservative management is an appropriate initial strategy, even moderate patients who are inclined to surgery are advocated for a period of conservative treatment. A variety of conservative treatments are advocated, including medications, bed rest, epidural steroid injections, physical therapy, therapeutic exercises, and combinations thereof [14]. Exercise therapy is a fundamental component of physical therapy that encourages self-management, aims to improve spine flexibility or mobility, and combat the physical and psychological effects of maladjustment related to pain and functional limitations, and it can be performed at a low cost and continued at home [15]. Previous meta-analyses of physical therapy in LSS patients reported that exercise therapy has better short-term outcomes for disability and back and leg pain than no exercise therapy [16], and the use of exercise alone or in combination with other non-pharmacological therapies was also recommended by Danish, US, and UK guidelines [17].

Little is known about the use of exercise therapy in patients with DLSS or the factors associated with exercise therapy, and few studies have focused on the impact of T2D on the prognosis of exercise therapy. Therefore, this study aimed to investigate the prognosis of exercise therapy for symptomatic DLSS in patients with T2D compared to a propensity score-matched control group and to analyze the association between symptomatic DLSS and T2D.

## Methods

### Subjects

**Fig. 1:**
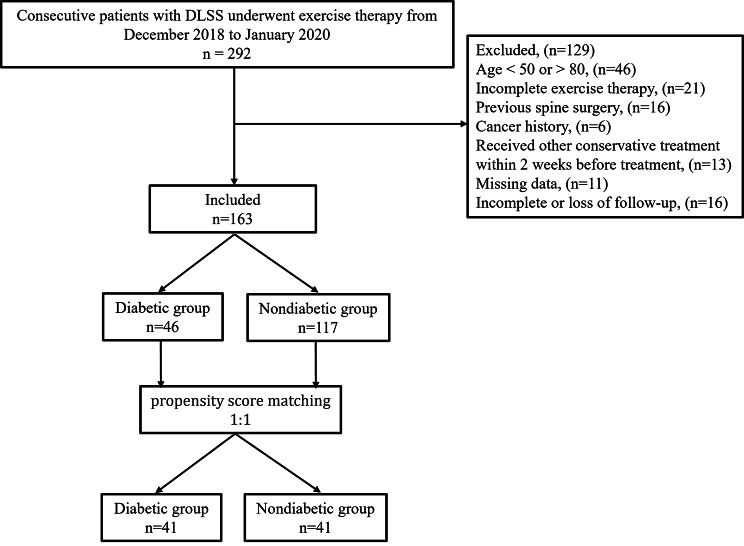
Flow chart used for patient enrollment and propensity score matching

This retrospective cohort study was approved by the Ethics Committee of Our Hospital. And all methods were carried out by the relevant guidelines and regulations. Patients with radicular DLSS visiting the outpatient Orthopedic Medicine at our hospital from December 2018 to January 2020 were enrolled based on the following eligibility criteria (Fig. 1): (1) Age from 50 to 80 years, neurogenic intermittent claudication; (2) Narrowed lumbar spinal canal, nerve root canal or intervertebral foramen confirmed by MRI; (3) Previously diagnosed as T2D and experienced endocrinologists excluded peripheral vascular and peripheral neuropathy of diabetes according to symptoms, vascular ultrasound or physical examination results. T2D was diagnosed based on the American Diabetes Association criteria [18] of FPG (fasting plasma glucose) > = 126 mg/dL (7.0 mmol/L), 2-h PG (2-h plasma glucose) > = 200 mg/dL (11.1 mmol/L) during OGTT (oral glucose tolerance test), HbA1c (glycated hemoglobin) > = 6.5%, or random plasma glucose > = 200 mg/dL (11.1 mmol/L) in patients with typical symptoms of hyperglycemia or hyperglycemic crisis. (4) those who have not received any conservative treatment within 2 weeks before treatment. And patients meeting any of the following criteria were excluded: (1) patients with loss of bowel or bladder control, cervical spondylosis, thoracic spinal stenosis, significant scoliosis; (2) Previous spine surgery, severe spinal spondylolisthesis, severe osteoporosis, metastasis to the vertebrae and comorbid infectious diseases; (3) patients with severe comorbidity that increased the risk to the patients or interfered with the assessment of the study (4) patients with cognitive changes or poor compliance.

### Clinical data

A total of 163 DLSS patients including 46 patients with T2D and 117 without T2D were included in this study. The age, sex, height, weight, body mass index (BMI), complications, duration of diabetes, FPG, HbA1c, polyneuropathy, insulin dependence, and other data of all patients were recorded.

Data were collected at baseline, 6 weeks, and 12 weeks after exercise therapy, Primary outcome measures were leg pain intensity (11-point Numerical Rating Scale, NRS) and low back disability (Oswestry Disability Index, ODI) [19]. The second outcome included low back intensity (NRS), self-paced walking test (SPWT), and the validated Chinese version of SF-36 BP (36-Item Short-Form Health Survey Bodily Pain) and PF (Physical Function) [20], Stenosis Bothersomeness Index (SBI) [21], Tampa Scale of Kinesiophobia (TSK-11) [22]and Beck Depression Inventory (BDI-II) [23]. For detailed analyses, we compared the proportions of patients achieving a minimum clinically important difference (MCID) for ODI and NRS. Based on the previous literature [24], the MCID for NRS in DLSS is between 1.25 and 1.5, and the MCID of ODI in a population receiving nonsurgical management for DLSS is 5% points.

### Imaging analysis

Magnetic resonance imaging (MRI) of the lumbar spine was performed to evaluate the levels, locations, and severity of the stenosis with a 3.0T system (Siemens Medical Solutions, Erlangen, Germany) with participants in the supine position. Imaging protocols included the acquisition of axial and sagittal T2-weighted images from T12 to the sacrum (repetition time/echo time, 3500/106; matrix size, 208*320), with a slice thickness of 4 mm.

The cross-sectional areas (CSA) of the dura sac in the axial plane on T2-weighted MRI images were used to classify the severity of spinal stenosis (mild, > 100 mm2; moderate, 76–100 mm2; severe, 75 mm2) [25].

Paraspinal muscles are the main source to maintain spinal stability, of which the multifidus muscle (MF) is particularly important for maintaining the stability of the lumbar spine due to its anatomical structure and morphological characteristics [26]. MF degeneration, including reduced volume, increased fatty infiltration, and bilateral muscle asymmetry, has a close association with LSS [27]. The MF measurements of interest included the CSA, functional CSA (FCSA, cm^2^) (i.e., the area of lean muscle tissue excluding fatty infiltration), and the ratio of FCSA to CSA (FCSA/CSA) at the L4-L5 segment as an indicator of muscle fat infiltration, was evaluated at the superior endplate level of L5 using the thresholding technique in ImageJ software (version 1.5, National Institutes of Health, Bethesda, Maryland, USA). To eliminate biases caused by differences in patient build, the relative CSA (RCSA, cm^2^) was calculated by dividing the muscle CSA by the CSA of the L5 vertebral superior endplate at the corresponding level (Fig. S1). The MF asymmetry in CSA was calculated based on the following formulae: [(L - S)/L)] *100, where L is the larger side and S is the smaller side. The above data were measured by two orthopedic spine surgeons and reached a consensus for all patients. The intraclass correlation coefficients (ICC) were calculated to access the interobserver reliability before statistical analysis.

### Supervised exercise therapy

**Fig. 2:**
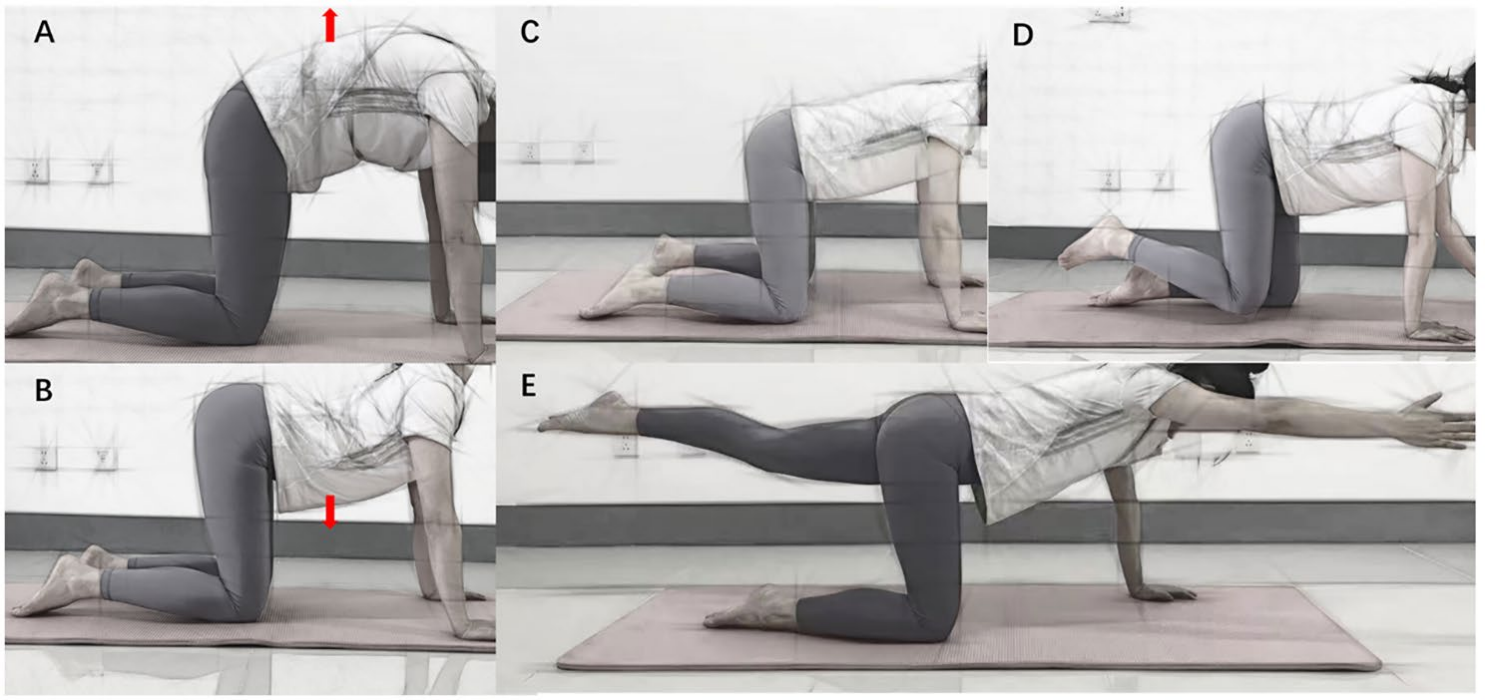
Lumbar stabilization exercises: A, B: cat-camel; C, D, E:b ird-dog

All patients received therapeutic exercises 3 times a week for 6 weeks, which included lumbar flexion, motor control exercises, lumbar stabilization, and walking under the supervision of a trained physical therapist. Flexion-based exercises, also known as William’s flexion exercises, have long been considered a standard treatment for lumbar spinal stenosis patients, and include single and double knee-to-chest exercises, hip flexor stretch, and squat, performed bilaterally and lasted approximately 40 min including 10 min warm-up (lower extremity stretching exercises), 20–30 min period of strength exercises with three sets of 10–12 repetitions. Motor control exercises are designed to improve the function of specific muscles in the low back region as well as posture and movement control, performed for three sets of 12–16 repetitions each, with two minutes rest periods between sets and exercises. Lumbar stabilization exercises include core stability exercises as well as spinal segmental stabilization exercises. Core stability exercises include the plank, lying straight leg raise, and “bird dog” to train neutral spine and imbalance control; spinal segmental stabilization exercises include “cat-camel” to train muscle strength in the deeper layers of the spine (Fig. 2, demonstrated by our physical therapist, maintaining equilibrium for 12–16 s for each, with a 6-second break between repetitions for approximately 40 min). Participants are also encouraged to perform exercise sessions at home 2 times per week according to a given program provided in the patient educational material. During the 6-week treatment, patients were allowed to continue taking their previously prescribed medications for DLSS, but could not change the type or dosage of those medications. Besides that, patients are encouraged to renew contact with their doctors to ensure medical compliance.

### Matching and statistical analysis

To minimize differences in baseline characteristics and confounding variables for T2D, a propensity score matching was performed using a 1:1 nearest neighbor matching algorithm without replacement, with a caliper width of 0.2 for the standard deviation of the logit. Factors including age, sex, BMI, symptom Duration, stenosis level, stenosis severity, ODI, NRS of low back pain, leg pain, SPWT, TSK-11, BDI, SBI, and SF-36 physical functioning, bodily pain at baseline were included in the logistic regression model. After matching, the standardized mean difference (SMD) was used to evaluate the balance of baseline variables. SMD < 0.20 between matching pairs reflects an acceptable balance. Data were presented as mean ± SD for continuous or frequencies for categorical variables. In the complete cohort, using the Mann-Whitney U test for nonparametric variables, Student’s t-test for parametric variables, and the χ2 test. Match pairs were analyzed using paired t-test for continuous variables, McNemar’s test for dichotomous variables, and Wilcoxon signed-rank test for ordinal categorical variables.

The Shapiro-Wilk test was used to determine whether the data had a normal distribution and whether to perform a logarithmic transformation on the skewed variables or use a nonparametric statistical test for analysis. Mean differences and percent differences at different time points among groups were investigated using repeated measurement analysis of variance (ANOVA) with the factors of time and group, Mauchly’s test of sphericity was used to judge whether there were relations among the repeated measures data. If any (P < 0.05), data were analyzed by multivariate analysis of variance or correction results of Greenhouse-Geisser. Then the Bonferroni t-test was used for pairwise comparisons of each group. All analyses were conducted in SPSS Statistics version 26.0. (IBM, Armonk, NY). Statistical significance was defined as p < 0.05 based on a 2-sided hypothesis test.

## Results

From December 2018 to January 2020, a total of 163 patients participated in this study, of which 46 and 117 participants were enrolled in the T2D and control groups, respectively (Fig. 1). Table 1 shows the baseline characteristics of participants in both groups. There were significant gender differences, and BMI **(**P = 0.048, and P = 0.024, respectively**).** The ICC between the two observers for the multifidus muscle measurements (the cross-sectional areas (CSA), functional FCSA, the relative CSA (RCSA), and the multifidus muscle asymmetry) was 0.86, 0.73, 0.75, and 0.78, respectively. Observers had good reliability with scoring head position and moderate reliability with scoring [28]. MRI measurement showed that the multifidus muscle fat infiltration and multifidus muscle asymmetry were significantly greater in DM-group (i.e., lower FCSA/CSA, 0.43 ± 0.12 vs. 0.51 ± 0.12, P = 0.031; 8.57 ± 5.01 vs. 6.40 ± 3.63, P = 0.016), no significant differences were noted between the groups in RCSA of the multifidus muscle. And there was no significant difference in the DLSS parameters such as symptom duration, stenosis level, stenosis locations, and stenosis severity. No significant difference in attendance rates was observed between the two groups, 78% of the DM group and 89% of the control group attended for complete attendance (> 80% of therapy attended).

**Table 1:** Comparisons in clinical data between type 2 diabetes mellitus group and control group before and after propensity score matching

	Before matching			After matching		
	Diabetic (n = 46)	Control (n = 117)	P Value	Diabetic (n = 41)	Control (n = 41)	P Value
**Age (years)**	69.8(6.9)	64.7(8.7)	0.062*	69.8(6.9)	69.2(8.7)	0.841*
**Gender, Female**	27(66)	59(50)	0.048^†^	25(61)	23(56)	0.654*
**Body Mass Index (kg/m²)**	29.2(4.3)	25.9(5.7)	0.024^‡^	29.2(4.3)	28.8(5.7)	0.866^†^
**Current Smoker (yes)**	7(15)	16(14)	0.595^†^	5(12)	8(20)	0.364^†^
**Duration of diabetes**			n.a.			n.a.
**< 10 years**	13(28)			12(29)		
**10–20 years**	19(41)			16(39)		
**> 20 years**	14(30)			13(32)		
**HbAlc (%)**			n.a.			n.a.
**≤ 6.5**	20(43)			18(44)		
**> 6.5**	26(57)			23(56)		
**Diabetes treatment**			n.a.			n.a.
**Drugs**	21(46)			19(46)		
**Insulin**	9(20)			7(17)		
**Drugs + Insulin**	8(17)			7(17)		
**none**	8(17)			8(20)		
**Co-Morbidities**,						
**Hypertension**	21(51)	53(45)	0.513^†^	19(46)	16(39)	0.503^†^
**Heart problem**	4(10)	9(8)	0.743^†^	3(7)	2(5)	1.000^†^
**Pulmonary problem**	2(5)	6(5)	1.000^†^	2(5)	3(7)	1.000^†^
**Stomach problem**	8(20)	15(13)	0.296^†^	6(15)	4(10)	0.500^†^
**Joint problem**	9(22)	25(21)	0.938^†^	7(17)	9(22)	0.577^†^
**Other**	4(10)	14(12)	1.000‡	2(5)	3(7)	1.000^†^
**Any Neurological Deficit (reflex, sensory or motor)**	12(27)	32(27)	0.870^†^	11(27)	8(20)	0.432^†^
**Symptom Duration (> 6 months)**	24(59)	71(61)	0.431^†^	22(57)	28(68)	0.174^†^
**Complete Attendance** ^**#**^	36	104	0.079^†^	32	36	0.240^†^
**MF RCSA**	0.42(0.09)	0.44(0.08)	0.189^‡^	0.41(0.08)	0.43(0.11)	0.126^‡^
**MF FCSA/CSA**	0.43(0.12)	0.51(0.12)	0.033^‡^	0.46(0.15)	0.49 (0.16)	0.038^‡^
**MF CSA asymmetry (%)**	8.57(5.01)	6.40(3.63)	0.016^‡^	8.09(4.88)	7.21(3.47)	0.021^*^
**Stenosis Level**			0.957^†^			0.957^†^
**L2 – L3**	3(7)	4(10)		3(7)	4(10)	
**L3 – L4**	6(15)	5(13)		6(15)	5(13)	
**L4 – L5**	21(51)	19(48)		21(51)	19(48)	
**L5 – S1**	11(27)	12(30)		11(27)	12(30)	
**Stenosis Levels (mod/severe)**			0.277^†^			0.463^†^
**None**	5(11)	13(11)		4(10)	3(7)	
**One**	21(46)	36(31)		19(46)	16(39)	
**Two**	13(28)	38(32)		11(27)	12(29)	
**Three or more**	7(15)	30(26)		7(17)	10(24)	
**Stenosis Locations**			0.852^†^			0.541^†^
**Central**	21(46)	56(48)		18(44)	23(56)	
**Lateral Recess**	17(37)	38(32)		15(37)	12(29)	
**Neuroforamen**	8(17)	23(20)		8(20)	6(15)	
**Stenosis Severity**			0.740^†^			0.258^†^
**Mild**	32(70)	74(59)		31(76)	24(59)	
**Moderate**	11(24)	31(29)		7(17)	12(29)	
**Severe**	3(7)	12(12)		3(7)	5(12)	
**NRS leg**	6.4(2.5)	6.9(2.1)	0.170^‡^	6.2(1.9)	6.3(2.1)	0.874^*^
**ODI**	35.7(11.8)	37.0(15.5)	0.031^‡^	35.2(12.1)	35.7(13.9)	0.831^‡^
**NRS back**	4.0(2.0)	3.8(1.4)	0.219^‡^	3.9(1.8)	3.8(1.2)	0.798^‡^
**SPWT**	457.3(230.4)	459.8(279.1)	0.418^‡^	464.4(221.4)	462.1(268.1)	0.812^*^
**SF-36 BP**	42.3(13.4)	42.8(16.6)	0.319^‡^	42.1(13.0)	42.2(15.8)	0.948^‡^
**SF-36 PF**	44.4(13.5)	47.4(14.5)	0.030^‡^	45.2(12.8)	46.3(14.9)	0.863^*^
**`SBI**	12.6(4.3)	11.8(4.3)	0.209^‡^	12.4(4.1)	11.5(4.2)	0.681^‡^
**TSK-11**	43.4(8.4)	41.4(11.5)	0.033^‡^	44.2(9.2)	44.8(14.2)	0.284^‡^
**BDI/**	7.5 (3.9)	5.9(4.1)	0.011^‡^	7.2 (3.6)	7.0(4.3)	0.928^*^

Values are mean ± SD or numbers (%). #: Complete attendance was defined as > 80% of therapy attended. Student’s t test or paired t-test;† χ2 test or McNemar’s test; ‡ Mann–Whitney U test; .n.a.: Not appliable; MF multifidus muscle; RCSA relative cross-sectional area, CSA cross-sectional area, FCSA/CSA ratio of functional cross-sectional area to total cross-sectional area. a: Student’s t test. b: χ2 test. c: Mann–Whitney U test

Propensity score matching yielded 41 well-matched T2D and control pairs. The mean standard differences for the covariates decreased from 0.532 to 0.071 after propensity score matching (good balance < 0.2). After propensity score matching of the two groups based on potentially confusing variables of T2D, including age, gender, and BMI, none of the variables were significantly different (Table 1). There was no significant difference in the outcome measurements between the T2D group the and control group at baseline.

### Prespecified outcome measurements

**Fig. 3:**
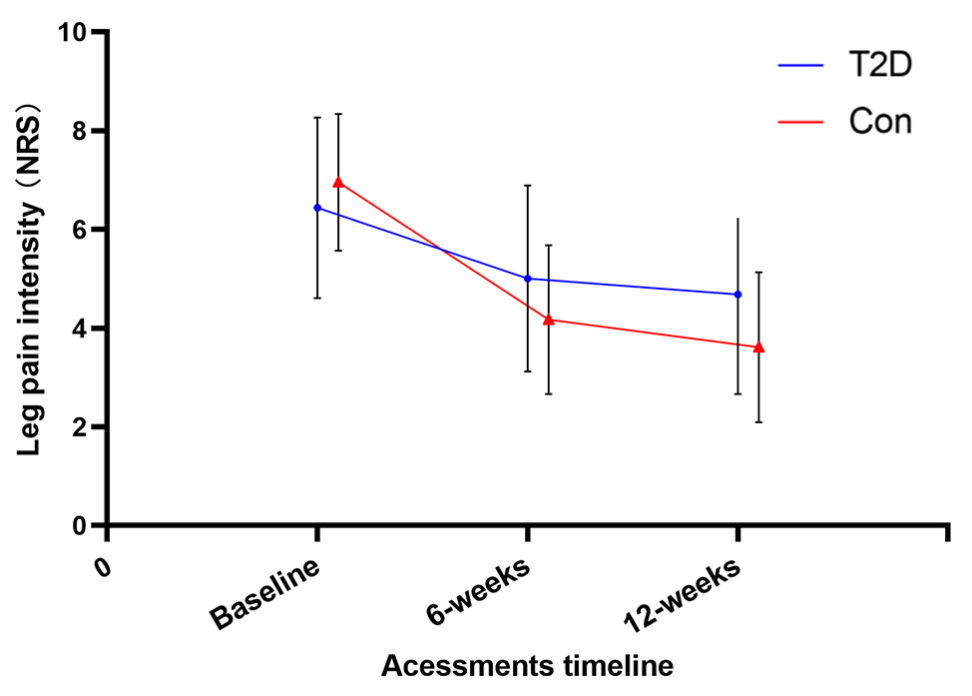
Leg pain intensity (NRS)

**Fig. 4:**
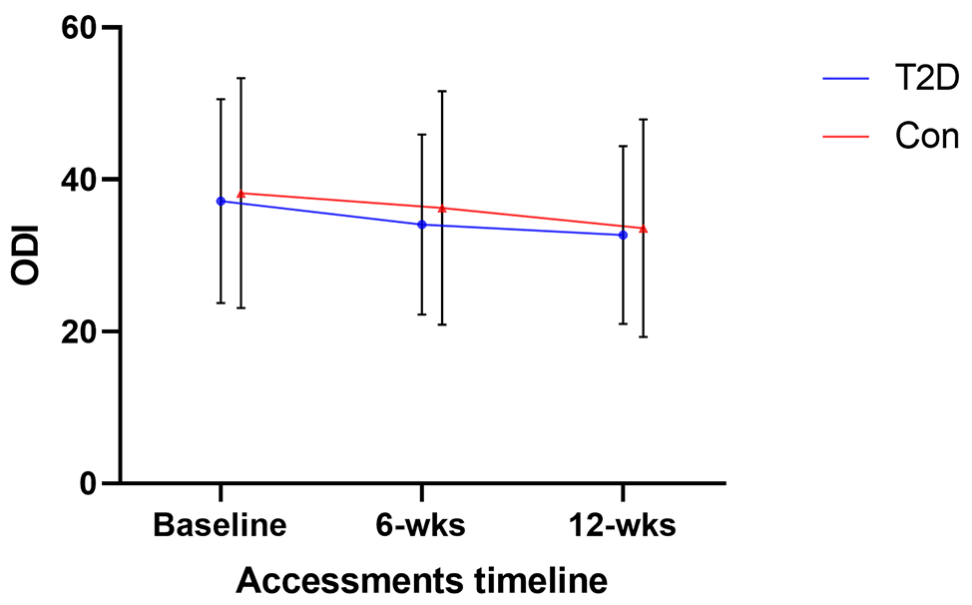
Oswestry Disability Index (ODI)

**Fig. 5:**
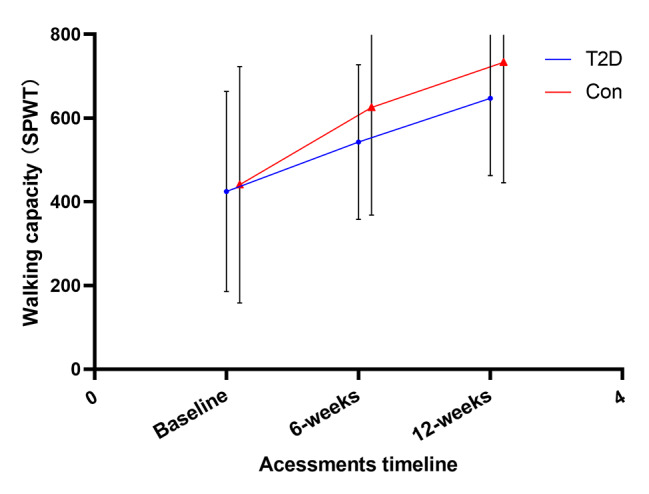
Walking capacity (SPWT)

No adverse events were reported during the 6-week therapeutic exercises. Forty-one pairs of patients were selected by propensity matching. Table 2 shows the results for time, group, and time group interaction effects of the prespecified outcome. After 6-week therapeutic exercises, patients with T2D achieved a lower improvement in leg pain at 6 weeks (NRS leg change, 1.21 ± 0.40 vs. 1.78 ± 0.52, P = 0.021) and 12 weeks (NRS leg change, 1.52 ± 0.92 vs. 2.18 ± 0.96, P = 0.007) above MCID, with a significant Group × Time interactions (F_1,80_ = 16.32, p < 0.001, ηp2 = 0.053). However, the two groups showed no difference in the improvement of ODI, although the sample had significant improvements at 6 weeks (ODI change 3.02 [95% CI, 2.08 to 2.77], P < 0.001) and 12 weeks ([ODI change 3.82 [95% CI, 4.03 to 4.90], P < 0.001), 46% of the patients achieved an MCID in the DM group, and 52% in the control group. And patients with T2D achieved a lower increase in walking capacity at 6 weeks (SPWT change, 129.28 ± 251.00 vs. 167.51 ± 149.83, P < 0.001) and 12 weeks (SPWT change, 220.17 ± 282.09 vs. 247.62 ± 345.40, P < 0.001), with a significant Group × Time interactions (F_1,80_ = 11.71, p = 0.027, ηp^2^ = 0.038). Figures 3 and 4, and 5 present between-group comparisons of leg pain intensity, low back disability, and walking capacity respectively.

**Table 2:** Comparisons in clinical outcome measures between type 2 diabetes mellitus group and control group after propensity score matching

Outcome Measures	Diabetic (n = 41) Mean + SD (95%CI)	Control (n = 41) Mean + SD (95%CI)	Main effect of time (p)	Main effect of group (p)	Group × time interaction (p)
Primary Outcomes					
NRS leg/10					
Baseline	6.2 ± 1.9 (5.9-7.0)	6.3 ± 2.1 (6.4–7.3)			
6 weeks	5.0 ± 1.7 (4.4–5.6)	4.2 ± 1.6 (3.6–4.6)	< 0.001	0.023	< 0.001
12 weeks	4.6 ± 1.9 (4.1–5.3)	3.7 ± 1.6 (3.0–4.0)			
**ODI /100**					
Baseline	35.2 ± 12.1 (32.9–41.4)	35.7 ± 13.9 (31.9–41.0)			
6 weeks	33.5 ± 10.2 (33.1–38.6)	34.6 ± 15.5 (29.4–38.6)	0.010	0.397	0.774
12 weeks	31.6 ± 9.9 (29.3–36.7)	33.0 ± 15.3 (27.8–36.8)			
**Second Outcomes**					
**NRS back/10**					
Baseline	3.9 ± 1.8 (3.3–4.6)	3.8 ± 1.2 (2.8–3.7)	0.031	0.155	0.122
6 weeks	2.9 ± 1.7 (2.3–3.4)	2.2 ± 1.2 (1.8–2.6)			
12 weeks	2.3 ± 1.2 (1.9–2.7)	1.9 ± 1.0 (1.6–2.2)			
**SPWT**					
Baseline	464.4 ± 221.4 (379.6–537.0)	462.1 ± 268.1 (351.3-529.7)			
6 weeks	545.3 ± 178.2 (478.0-612.7)	651.4 ± 246.0 (544.0-706.3)	< 0.001	0.042	0.027
12 weeks	612.7 ± 189.1 (552.3-682.3)	776.5 ± 269.3 (642.1-823.8)			
**SF-36 BP /100**					
Baseline	42.1 ± 13.0 (38.1–46.5)	42.2 ± 15.8 (37.6–48)			
6 weeks	47.6 ± 12.2 (43.8–51.5)	48.2 ± 16.0 (43.2–53.3)	0.363	0.134	0.291
12 weeks	49.4 ± 11.6 (45.7–53.1)	52.5 ± 16.2 (47.4–57.6)			
**SF-36 PF/100**					
Baseline	45.2 ± 12.8 (40.1–48.6)	46.3 ± 14.9 (42.8–52.0)			
6 weeks	47.1 ± 13.8 (42.7–51.4)	50.0 ± 14.7 (45.3–54.6)	0.247	0.182	0.121
12 weeks	49.9 ± 13.5 (45.7–54.2)	53.7 ± 15.3 (48.9–58.6)			
**SBI/24**					
Baseline	12.4 ± 4.1 (11.2–12.9)	11.5 ± 4.2 (10.4–13.2)			
6 weeks	9.5 ± 3.8 (8.3–0.7)	8.7 ± 4.7 (7.3–10.2)	0.032	0.098	0.166
12 weeks	8.0 ± 3.8 (6.8–9.2)	6.7 ± 3.3 (5.7–7.8)			
**TSK-11/68**					
Baseline	44.2 ± 9.2 (40.7–46.0)	44.8 ± 14.2 (37.7–45)			
6 weeks	41.0 ± 7.8 (38.5–43.4)	38.3 ± 11.9 (34.6–42.1)	0.128	0.144	0.453
12 weeks	39.6 ± 7.7 (37.2–42.0)	36.8 ± 12.7 (32.8–40.8)			
**BDI/63**					
Baseline	7.2 ± 3.6 (6.3–8.7)	7.0 ± 4.3 (4.6 to 7.2)			
6 weeks	5.9 ± 4.0 (4.5–6.9)	5.7 ± 4.3 (4.1 to 6.3)	0.087	0.106	0.051
12 weeks	5.3 ± 3.6 (4.3–6.6)	5.0 ± 3.9 (3.8 to 6.2)			

SD standard deviation, CI confidence interval. **NRS**: 11-point Numerical Rating Scale, the NRS leg pain and back pain is an 11-point scale where the endpoints are the extremes of “no pain” and “pain as bad as it could be”; **ODI**: Oswestry Disability Index, the ODI is scored from 0–100 with higher numbers indicating greater disability; **SPWT**: self-paced walking test, the SPWT is measured as the distance a person can walk continuously on a flat surface at a self-selected pace until being forced to stop because of symptoms of LSS, including neurogenic claudication and/or low back pain, up to a limit of 30 min; SF-36 BP/PF: **SBI**: Stenosis Bothersomeness Index, a 0–24 scale, with higher scores indicating more bothersome symptoms. The Index is calculated as the sum of separate 0–6 bothersomeness ratings for leg symptoms of pain, weakness, numbness, and pain after walking. **TSK-11**: The Tampa Scale for Kinesiophobia, TSK-11 is a widely used questionnaire to assess fear of movement. The TSK consists of 17 items, each rated on a 4-point Likert scale ranging from “strongly disagree “to strongly agree”. Four items are inversely phrased. The total sum has a minimum score of 17 (low fear) and a maximum of 68 (high fear); **BDI**: Beck Depression Index, BDI is a 21-item, self-report rating inventory that measures characteristic attitudes and symptoms of depression. *Statistically significant difference between groups

Back pain intensity after therapeutic exercises showed a significant time-effect improvement, achieved an MCID in 72% of patients in the DM group, and 78% in the control group, but showed no Group × Time interactions. Numerous psychological variables showed an improvement in both groups over time, including SBI, fear-avoidance behavior (TSK-11,) and level of anxiety and depression (BDI), but no group significant difference was found (all p > 0.05).

## Discussion

Previous Spine Patient-Reported Outcomes Related Trials (SPORT) have demonstrated that nonsurgical treatment methods provide greater short-term improvement in symptoms, physical function, and walking capacity in patients with LSS [29, 30], however, there have been few studies on the effectiveness of exercise therapy in patients with T2D. Due to the strong connection between T2D and lumbar spine disorders [9], this propensity scores matching study aimed to compare the effectiveness of a 6-week exercise-based therapy for symptomatic DLSS in patients with T2D compared to nondiabetic patients. Most outcomes showed a similar trend with improvements seen at the baseline assessment, reflecting therapeutic exercises’ potential benefits. In addition, the results showed no significant difference in low back disability improvement between the two groups, however, leg pain intensity and walking capacity showed clinically and statistically significant Group×Time interactions.

Flexion-based lumbar stabilization exercises can improve paravertebral muscle activation, trunk muscle strength, endurance, and muscle cross-sectional area, to improve lumbar spine stability and coordination, reducing the risk of nerve compression and improving pain, disability, and other symptoms in LSS patients [31, 32]. During the 12-week exercise therapy, 86% of patients had good treatment compliance, attending scheduled therapy regularly, in addition, the patients with T2D did not show difficulty in performing exercise movements compared to the controls. As one of the primary goals of treatments for DLSS is to improve pain-related disability, an especially limited walking capacity which is one of the main reasons patients with LSS seek medical care [33], however, our results found a greater reduction in leg pain intensity and a better-improved performance of walking capacity in nondiabetic patients, with significant Group × Time interactions (F_1,80_ = 16.32, p < 0.001, ηp2 = 0.053; F_1,80_ = 11.71, p = 0.036, ηp^2^ = 0.038). Meanwhile, no significant difference in the improvement of ODI between the two groups, the possible reason is that ODI is difficult to sufficiently evaluate patients with mild to moderate disability [34, 35].

Considering the close association of the paraspinal muscles and DLSS [27], the MF degeneration including muscle volume (RCSA), fatty infiltration (FCSA/CSA), and bilateral muscle asymmetry was compared between the two groups. After matching, the T2D group still showed a significantly higher multifidus muscle fat infiltration, and multifidus muscle asymmetry than the control group (0.36 ± 0.22 vs. 0.52 ± 0.23, P = 0.03; 8.57 ± 5.01 vs. 6.40 ± 3.63, P = 0.026), and no significant differences were noted between the groups in RCSA of the multifidus muscle. Researchers had found that MF degeneration (i.e. reduced volume, increased fatty infiltration, and bilateral muscle asymmetry) is correlated with lower functional status as indicated by the ODI in patients diagnosed with LSS [36], multifidus degeneration could further aggravate the instability of the lumbar spine, resulting in hyperplasia of intraspinal ligaments and bones, which could negatively affect the outcome of exercise therapy for DLSS, indicating a strengthening program of lumber extensor muscle could help prevent amyotrophy and lumbar degeneration. And aberrant MF morphometry/function was related to the negative clinical outcomes of patients after various conservative treatments [37], which may contribute to the inferior therapeutic effect of patients with T2D.

Diabetes has been considered a possible factor aggravating lumbar disease [38], and studies have shown that patients with diabetes received more lumbar surgery and had more postoperative complications than non-diabetic patients [11–13]. In our study, patients with D2D showed worse results on exercise therapy than control patients, which may have been related to coexisting diabetic neuropathy causing nerve susceptibility to ischemic changes [39, 40] and increased sensitivity to mechanical stimulation [41, 42] may be an important factor contributing to this result.

The present findings provided new insights for conservative treatment for DLSS patients with T2D, clinicians need to consider the negative impact of diabetes on the efficacy of exercise therapy, to choose the most appropriate therapy for each patient. There were still limitations in this study, including the fact that it was a retrospective single-center study with the possibility of incomplete data collection, a limited data source, and a selection bias. We used propensity score matching to avoid these biases. In our study, neurophysiological study such as nerve conduction velocity test was not tested in all patients to evaluate diabetic neuropathy, and other complications, such as microangiopathy, diabetes duration, and insulin dependence that could affect the prognosis of diabetic patients were not fully controlled for in this study. To further clarify the efficacy of exercise therapy in patients with diabetes and lumbar spinal stenosis, a prospective study controlling these confounding factors is required, and future trials will require strictly supervised exercise therapy. The follow-up period in the current study was insufficient to evaluate long-term results and the effect of a 6-week therapeutic exercise intervention on reducing the possibility of surgery in patients with T2D. More trials with a longer follow-up period were required to determine the efficacy of therapeutic exercises on patients with lumbar spinal stenosis. Although the therapeutic exercises in this study were only done for 6 weeks, patients with diabetes may require more long-term therapy to improve the treatment effect. Furthermore, psychological factors such as pain catastrophes and fear-avoidance beliefs did not improve significantly at 6 and 12 weeks. Future research should focus on whether psychological factors can help DLSS patients maintain the treatment effect and improve clinical outcomes during the disease’s natural duration.

## Conclusion

Six-week therapeutic exercises have an inferior effect on DLSS patients with T2D compared to propensity score-matched controls, especially in the improvement of leg pain and walking capacity. The results of this study provide new evidence about exercise therapy as a viable nonsurgical treatment option for patients with LSS and improve the understanding of DLSS treatment in diabetes patients.

## Electronic supplementary material

Below is the link to the electronic supplementary material.

Supplementary Material 1

## Data Availability

The datasets used and/or analyzed during the current study are available from the corresponding author upon reasonable request.

## List of abbreviations

DLSS: Degenerative lumbar spinal stenosis
T2D: Type 2 diabetes mellitus
NRS: Numerical Rating Scale
ODI: Oswestry Disability Index
SPWT: Self-paced walking test
MF: Multifidus muscle
CSA: Cross-sectional areas
