# Supplementary material for: Does type 2 diabetes affect the efficacy of therapeutic exercises for degenerative lumbar spinal stenosis?
Source: BMC Musculoskelet Disord. 2023 Mar 16;24:198. doi: 10.1186/s12891-023-06305-0 (PMC10018869; doi:10.1186/s12891-023-06305-0)
Supplement: Supplementary file 1 — Supplementary Material 1 [file 12891_2023_6305_MOESM1_ESM.docx]

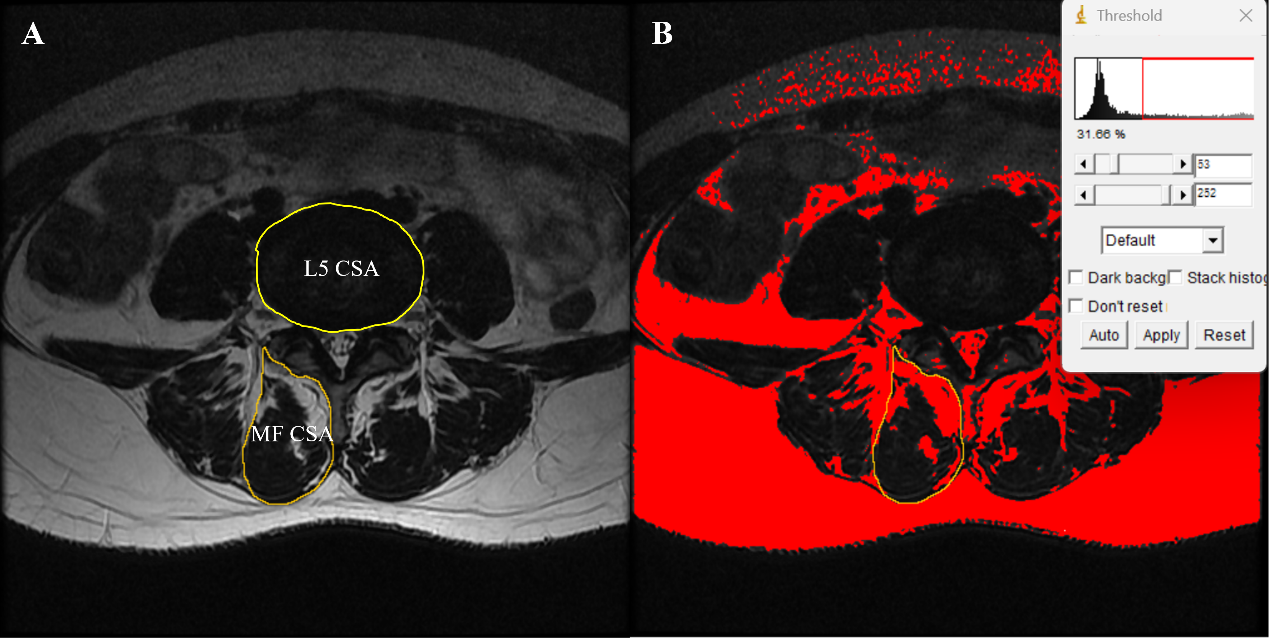


Fig. S1. Measurement of the multifidus muscle (MF): A. Multifidus (MF) cross-sectional area and the total cross-sectional area of the L5 vertebra were obtained bilaterally, on T2-weighted axial images; B.The functional cross-sectional area of muscle (FCSA, the area of lean muscle tissue excluding fatty inﬁltration) were measured by using the thresholding technique in ImageJ software. RCSA = summation of bilateral FCSA/L5 CSA (cross-sectional area of the vertebral body). Asymmetry= [(L - S)/L)] *100, where L is the larger side and S is the smaller side.s
